# Supplementary material for: Complexome profiling of the Chlamydomonas psb28 mutant reveals TEF5 as an early PSII assembly factor
Source: Plant Cell. 2025 Mar 17;37(6):koaf055. doi: 10.1093/plcell/koaf055 (PMC12164590; doi:10.1093/plcell/koaf055)
Supplement: koaf055_Supplementary_Data [file koaf055_supplementary_data.zip › psb28_interactive.zip!/plots/intensity/CYN26-2_Cre03.g174750.t1.2.html]

|  |  |
| --- | --- |
| **Identifier** | Cre03.g174750 |
| **Trivial name** | CYN26-2 |
| **Euclidean distance** | 1776.81 |
| **Average intensity sum (WT)** | 123.5 || **Average intensity sum (Mut)** | 89.6 |
| **Adjusted euclidean distance** | 14.39 |
| **MapMan annotation** | cell.cycle.peptidylprolyl isomerase protein.folding.immunophilins (IMM).cyclophilins |
| **p-value** (Welch test) of intensity sums | 0.72001 |
